# Diurnal Fluctuations of Orexin-A and -B in Cynomolgus Monkey Cerebrospinal Fluid Determined by a Novel Analytical Method Using Antiadsorptive Additive Treatment Followed by Nanoflow Liquid Chromatography–High-Resolution Mass Spectrometry

**DOI:** 10.1021/acschemneuro.2c00370

**Published:** 2023-01-31

**Authors:** Naohiro Narita, Ryuji Yamada, Masaaki Kakehi, Haruhide Kimura

**Affiliations:** † Drug Metabolism and Pharmacokinetics Laboratory, Research, Takeda Pharmaceutical Company Limited, 26-1 Muraoka-Higashi 2-chome, Fujisawa, Kanagawa 251-8555, Japan; ‡ Neuroscience Drug Discovery Unit, Research, Takeda Pharmaceutical Company Limited, 26-1 Muraoka-Higashi 2-chome, Fujisawa, Kanagawa 251-8555, Japan

**Keywords:** orexin, hypocretin, diurnal fluctuation, cerebrospinal fluid, liquid chromatography−mass spectrometry, narcolepsy

## Abstract

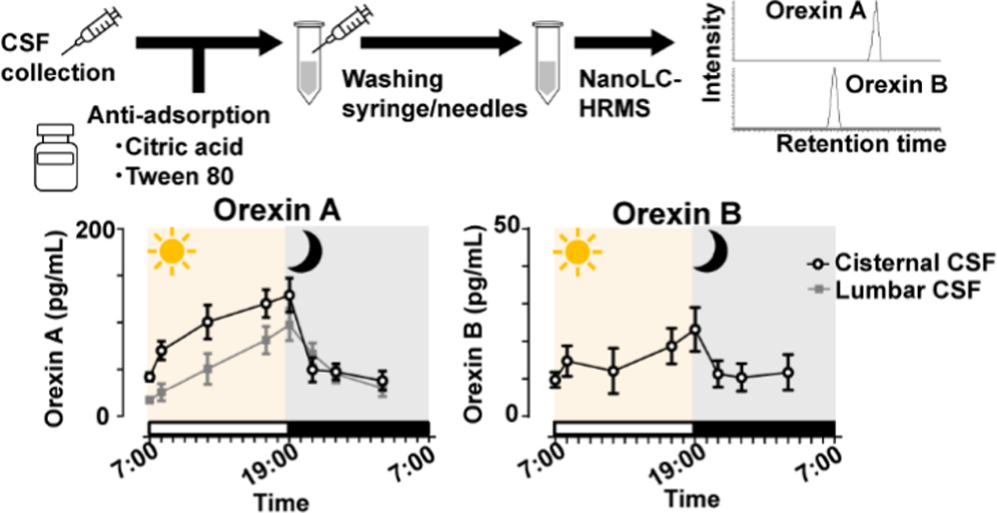

Orexin-A (OXA) and -B (OXB) are involved in the regulation of multiple physiological functions including the sleep–wake states; therefore, it is critical to monitor their levels under various conditions. Unfortunately, the widely used radioimmunoassay has insufficient specificity for OXA. Although liquid chromatography–tandem mass spectrometry (LC–MS/MS) has higher specificity for OXA, previously reported OXA levels in human cerebrospinal fluid (CSF) measured using this technique are still inconsistent. Moreover, to the best of our knowledge, OXB has not been detected in the CSF. In this study, we established a novel method for OXA and OXB measurement. We noticed that OXA and OXB in the CSF was sticky; thus, citric acid and Tween 80 were used to prevent their nonspecific binding. Then, highly specific and sensitive nanoflow liquid chromatography–high-resolution mass spectrometry (nanoLC-HRMS) was used to measure OXA and OXB levels. Evaluation of the diurnal fluctuations of OXA and OXB in cisternal and lumbar CSF samples from cynomolgus monkeys revealed a sharp increase in the early light period, followed by a gradual increase to the maximum levels at the end of the light period, and then a sharp drop to the minimum levels during the early dark period. OXB levels were lower than OXA levels in cisternal CSF. Although basal OXA levels in individual monkeys showed substantial variations, the ratios between the maximum and minimum OXA levels of each monkey were similar. Our method for accurate OXA and OXB measurement should help improve our knowledge of orexin biology.

## Introduction

Orexin-A (OXA) and orexin-B (OXB) (also called hypocretin-1 and hypocretin-2, respectively) are released from orexin neurons in the hypothalamus. They are involved in various physiological functions including regulation of the sleep–wake states.^1−3^ OXA and OXB are produced by proteolytic cleavage and post-translational modifications (PTMs) of a common precursor protein, prepro-orexin.^1,4^ OXA is a 33-amino acid peptide with N-terminal pyroglutamylation, amidation of the C-terminal leucine, and two intramolecular disulfide bridges. OXB is a 28-amino acid peptide with a C-terminal sequence similar to that of OXA including C-terminal amidation, while its N-terminus has no modifications. OXA and OXB are ligands of two G-protein-coupled receptors, orexin 1 receptor (OX1R) and orexin 2 receptor (OX2R).^1^ Loss of orexin neurons and the resultant orexin neuropeptide reduction are associated with narcolepsy type 1 (NT1), which is a severe neurological disorder characterized by multiple symptoms including hypersomnia, cataplexy, disturbed nighttime sleep, hypnagogic/hypnopompic hallucinations and sleep paralysis.^5−7^ Because OX2R knockout (KO) mice show clear narcolepsy-like phenotypes and OX1R KO mice do not, OX2R-selective agonists are a promising option for treating NT1.^3,8^ Recently, two compounds (YNT-185 and TAK-925) have been discovered as novel OX2R-selective agonists.^9,10^ As NT1 is related to selective loss of orexin neurons, the OXA level in the cerebrospinal fluid (CSF) has been used as a diagnostic biomarker for NT1.^11,12^ The OXA level in the CSF has been measured by competitive radioimmunoassay (RIA).^11,12^ However, a recent report suggested that more than 90% of the signal detected by RIA reflects biologically inactive OXA-related metabolites.^13^ Detailed analysis has shown that antibodies (Abs) employed in the RIA methods, including Phoenix Ab used for diagnostic testing, could capture not only OXA but also its metabolites, possibly C-terminal-truncated OXA.^13^ Therefore, improved methods to precisely measure OXA should be established. Sandwich immunoassay using two Abs that bind to different sites on the ligand may have higher specificity toward authentic bioactive OXA compared with RIA using a single Ab. However, no sandwich immunoassays for OXA, including electrochemiluminescence (ECL), have fully demonstrated specificity for authentic OXA.^14^ Liquid chromatography–tandem mass spectrometry (LC–MS/MS) is also a promising method for measuring OXA with high specificity because its detection is based on differences in the mass-to-charge ratio (*m*/*z*). Theoretically, LC–MS/MS could distinguish OXA from its metabolites like truncated OXA unless the metabolites give remarkably similar *m*/*z* to OXA; for example, deamidation at the C-terminal could show quite similar *m*/*z* to that of OXA (Δ*m*/*z* = −0.984). Thus far, three LC–MS/MS methods have been reported for measuring OXA in human CSF.^15−17^ However, the median OXA concentrations from those studies differed from each other by factors as high as 10 for healthy human subjects. It is well known that some peptides adsorb to the surface of materials, such as syringes, needles, and sample tubes.^18^ Therefore, the nonspecific binding of OXA could be a possible reason for this discrepancy. Moreover, reported methods using conventional LC–MS/MS may not be able to discriminate OXA from its metabolites with slight modification such as deamidation. Furthermore, no LC–MS/MS methods have detected OXB in the CSF, although OXA and OXB are produced in the same amount by the cleavage of prepro-orexin.^17^ Thus, little is known about the distribution and physiological functions of OXB.^1^ A combination of nanoflow LC and high-resolution mass spectrometry (HRMS) is a promising approach to address this problem because of their higher specificity, sensitivity, and separation efficiency compared to conventional LC–MS/MS.

Characterizing the diurnal fluctuation of orexins is critical for understanding orexin signaling. Immunoassays (e.g., RIA and ECL) have been used to measure diurnal fluctuations of OXA in cisternal CSF samples collected from not only nocturnal animals like mice and rats but also diurnal animals like dogs, rhesus monkeys, and squirrel monkeys.^14,19,20^ The results showed a similar pattern among these animals: a higher level of OXA during the active period and a lower level during the resting period that dropped below 50% of the peak levels. In comparison, human OXA levels in lumbar CSF display smaller fluctuations, within ±10% from the 24 h average.^21^ This difference could be due to (1) limited specificity of the RIA and ECL methods for OXA, (2) species difference, and (3) the site of CSF correction (cisternal vs lumbar). More specific methods for OXA measurement would help to clarify these questions.

In this paper, we report a novel method that uses additives to prevent the nonspecific binding of OXA and OXB, and nanoflow LC coupled with HRMS (nanoLC-HRMS) for highly specific and sensitive detection of these two peptides. Using this method, we examined the diurnal fluctuations of both OXA and OXB in cisternal and lumbar CSF samples collected from cynomolgus monkeys.

## Results and Discussion

### Strategy for Quantitation of OXA and OXB in the CSF

We took advantage of the nanoLC-HRMS approach to precisely measure OXA and OXB. The nanoflow LC system provides higher sensitivity and better separation than conventional high-performance liquid chromatography. Survivor-selected ion monitoring (SIM) analysis using HRMS enables highly sensitive and specific detection of the target analytes.^22^ Nonspecific binding of peptides to syringes and sample tubes is a serious issue in bioanalytical assays, especially when using CSF which lacks proteins.^17,23^ We assessed the magnitude of nonspecific binding of orexins and evaluated antiadsorptive agents to overcome the nonspecific binding.

### Analytical Validation of the nanoLC-HRMS Method

**Figure 1:**
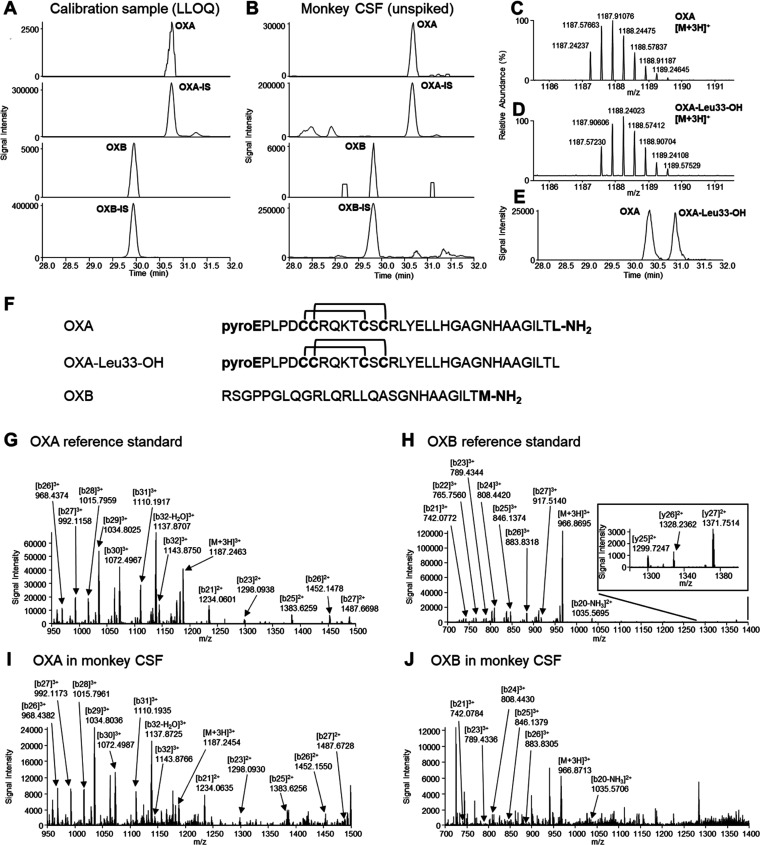
Representative chromatograms of OXA, OXB, and ISs from (A) calibration sample at LLOQ 2.5 pg/mL and (B) a cisternal CSF sample at 7:00. The peaks were detected with no closely eluting interferences. Isotope distribution of the [M + 3H]^3+^ charged reference standards for (C) OXA and (D) OXA-Leu33-OH. (E) LC separation of OXA and OXA-Leu33-OH as a putative metabolite (extracted ion chromatogram at *m*/*z* 1187.57 to 1187.58). Sufficient LC separation was required for the specific detection of each one due to their similar isotope distributions. (F) Amino acid sequences of OXA, OXA-Leu33-OH, and OXB. Product ion spectra of the [M + 3H]^3+^ precursors in parallel reaction monitoring (PRM) of reference standards for (G) OXA and (H) OXB at 500 pg/mL, and (I) OXA and (J) OXB in a monkey cisternal CSF sample at 17:00. Characteristic ions were assigned based on the *m*/*z* values of monoisotopic ions.

The established nanoLC-HRMS method seemed to have sufficient specificity and sensitivity for measuring OXA and OXB in monkey CSF (Figure 1A,B). The calibration curves were linear over the range of 2.5–250 pg/mL for both peptides. The correlation coefficients were within 0.9958–0.9987. Equivalent slopes of the surrogate and real matrices were observed for OXA (0.0038 and 0.0040, respectively) and OXB (0.0053 and 0.0054, respectively). The back-calculated concentrations of the calibration standards ranged from 91.4 to 109.6%. Reproducibility was evaluated by intraday and interday assays of spiked quality control (QC) in the surrogate matrix (SQC) and in the real matrix (RQC). The accuracy (percent mean relative error [% RE]) and precision (coefficient of variation [% CV]) in intraday and interday assays were within 9.3–2.0 and 14.0%, respectively (Table 1). The matrix effect was corrected using stable isotope-labeled OXA and OXB as internal standards (ISs). The unlabeled impurities in ISs had no impact on the quantitation of unlabeled orexins (Figure S1). Carry-over was assessed by injecting blank samples immediately after a calibration standard at the upper limit of quantification. The carry-over was lower than 20% of LLOQ for orexins and 5% for the ISs. The stability of OXA and OXB in the real matrix was evaluated using unspiked and spiked QC samples in triplicate. OXA and OXB were stable during three freeze–thaw cycles at −80 °C, at room temperature for 2 h, and at −80 °C for 60 days. The percent difference in QC samples for stability evaluation was within ±15% of the initial sample. The processed sample stability was evaluated using three replicates of real matrix (endogenous levels ranging from 93.7 to 179.5 pg/mL for OXA and from 14.9 to 26.6 pg/mL for OXB). The percent remaining after 48 h in an autosampler at 10 °C was within ±15% of the initial analysis. The result indicated that OXA and OXB were stable for at least 48 h in an autosampler at 10 °C. The preparation and storage of all CSF samples were conducted under the stable conditions described above. Compared to the reported LC–MS/MS method for OXA (LLOQ > 3.6 pg/mL), our method can measure OXA using only approximately 1/10 of the CSF sample volume and achieved lower LLOQ (2.5 pg/mL).^15−17^

**Table 1:** Analytical Validation Parameters of nanoLC-HRMS Method for OXA and OXB Measurement^a^

				intraday (*N* = 5)		interday (*N* = 15)	
analyte	matrix		theoretical concentration (pg/mL)	RE (%)	CV (%)	RE (%)	CV (%)
OXA	surrogate	LLOQ	2.5	–3.3	7.8	1.9	12.8
		LQC	7.5	–4.9	4.8	–1.5	6.6
		MQC	120	0.4	2.6	0.3	2.1
		HQC	200	0.6	1.8	–0.5	1.9
	real	LQC	57.7	–9.3	7.7	–8.8	14.0
		HQC	157.7	–0.8	5.0	0.3	6.4
OXB	surrogate	LLOQ	2.5	0.0	10.3	2.0	9.6
		LQC	7.5	0.1	4.6	–4.3	8.2
		MQC	120	–7.6	2.4	–3.3	2.7
		HQC	200	–2.1	3.3	–3.9	2.9
	real	LQC	28.2	–3.5	8.6	–5.6	9.7
		HQC	128.2	1.6	2.6	–4.0	5.4

a Intraday and interday assay precision (% CV) and accuracy (% RE) were evaluated in the surrogate matrix at 2.5 pg/mL (LLOQ), 7.5 pg/mL (lower quality control [LQC]), 120 pg/mL (middle quality control [MQC]), and 200 pg/mL (higher quality control [HQC]), and in real matrix spiked with orexins at 25 pg/mL (LQC) and 125 pg/mL (HQC).

### Assessment for Selective Detection of OXA and OXB in Monkey CSF

Selective detection of OXA and OXB in monkey CSF was further assessed based on retention time and characteristic product ions obtained by parallel reaction monitoring (PRM). The concentrations of OXA and OXB in the CSF sample used in this analysis were 179.5 and 26.6 pg/mL, respectively. The same product ions with reference standard OXA at 500 pg/mL were detected in monkey CSF (Figure 1G,H). Similar to reference standard OXB at 500 pg/mL, characteristic b-series ions for OXB were also detected in monkey CSF (Figure 1I,J). Several product ions with low signals, such as b22, b27, and y-series ions, obtained by 500 pg/mL of standard OXB were not detected in monkey CSF due to its low concentration of 26.6 pg/mL. These results further supported selective detection of OXA and OXB in monkey CSF.

### Separation of a Putative OXA Metabolite

OXA Abs used in RIA methods only recognize the N-terminal amino acid sequence.^13^ Thus, those methods cannot discriminate OXA from its metabolites that have an intact N-terminal region and a modified C-terminal region such as truncations. In contrast, the LC–MS/MS methods can theoretically separate OXA from truncated metabolites according to their detection principle. However, even these methods could not easily discriminate OXA from its metabolites with similar *m*/*z*, such as deamidation with Δ*m*/*z* < 1 due to multiple charges. A putative metabolite is an OXA-related molecule modified with deamidation at the C-terminal leucine, OXA-Leu33-OH (Figure 1F). Therefore, we analyzed OXA-Leu33-OH to confirm the high specificity of our nanoLC-HRMS method. Although similar isotope distributions were observed between OXA and OXA-Leu33-OH, the monoisotopic ion of OXA (*m*/*z* 1187.24237) was completely separated from that of OXA-Leu33-OH (*m*/*z* 1187.57230) using HRMS (Figure 1C,D). Moreover, by applying a high separation nanoflow LC system, we achieved sufficient LC separation between OXA and OXA-Leu33-OH (Figure 1E). Considering the high separation delivered from both HRMS and nanoflow LC, other OXA metabolites can be also separated in our method. OXA-Leu33-OH was not identified in monkey cisternal and lumbar CSF (Figure 1B); note that another peak following OXA should be observed, like Figure 1E, if OXA-Leu33-OH existed. The minor peaks around 31 min in monkey CSF were not identified as metabolites due to insufficient mass spectra (Figure 1B). The results suggested that the discrepancy in human CSF OXA levels measured by LC–MS/MS methods is not due to this metabolite.

### Overcoming Nonspecific Binding of Orexins during Sample Collection and Preparation

The recovery of spiked OXA and OXB at the final concentration of 100 pg/mL in neat monkey CSF was 41.6 and 40.2%, respectively, and these low values could be due to nonspecific binding and/or enzymatic degradation (Table 2). Detergents like Tween 80 were commonly used to prevent nonspecific binding. Acidification of samples can inhibit both nonspecific binding of peptides and enzymatic activity. To overcome those problems, a mixture of 20 mM citric acid and 0.1% (w/v) Tween 80 (final concentrations) was added to the monkey CSF. The treatment with 20 mM citric acid and 0.1% (w/v) Tween 80 improved the recoveries to 107.1% for OXA and 95.8% for OXB. Sufficient recovery was observed even after three tube transfers (OXA, 105.2%; OXB, 94.1%). Next, recovery during syringe transfer was assessed using the following procedure (Figure S2). A tube containing neat CSF spiked with the standards for OXA and OXB was prepared. Then, the spiked CSF was collected from the tube using a needle and a syringe. To the tube, 6 μL of 1M citric acid and 3 μL of 10% (w/v) Tween 80 were added, and the 300 μL of spiked CSF was returned from the syringe to the tube. The tube, the needle, and the syringe were rinsed by pipetting with CSF containing 20 mM citric acid and 0.1% (w/v) Tween 80 (final concentrations). The results demonstrated satisfactory recovery (OXA, 100.6%; OXB, 91.2%). Similar recovery was observed with the addition of 0.01% (v/w) Tween 80 alone (OXA, 106.1%; OXB, 81.4%). Thus, 0.01% (w/v) Tween 80 would be a sufficient additive to measure orexin levels in monkey CSF, and addition of citric acid had no negative impact on OXA and OXB measurement. These results supported that the improved recovery was not derived from the inactivation of degrading enzymes by acidification but rather from inhibition of nonspecific binding. Given the low recovery of orexins in neat CSF, a portion was considered to be adsorbed to the tubes, needles, and syringes before the addition of the antiadsorptive additives. The observed adequate recovery indicated that the antiadsorptive additives could not only prevent nonspecific binding of orexins during sample transfer but also recover orexins that were once adsorbed to syringes and tubes.

**Table 2:** Recovery during Syringe and Tube Transfer^a^

		recovery (%)	
additives to CSF	parameter	OXA	OXB
20 mM citric acid/0.1% tween 80	syringe and tube transfer	100.6 ± 9.4	91.2 ± 4.7
	tube transfer (3 times)	105.2 ± 12.1	94.1 ± 6.8
	spike recovery	107.1 ± 3.8	95.8 ± 1.2
none	spike recovery	41.6 ± 3.9	40.2 ± 3.5

a Recovery (%) of OXA and OXB (mean ± SD, *N* = 3). The addition of 20 mM citric acid and 0.1% (w/v) Tween 80 (final concentrations) prevented nonspecific binding of OXA and OXB.

### Diurnal Fluctuations of OXA and OXB Levels in Monkey CSF

**Figure 2:**
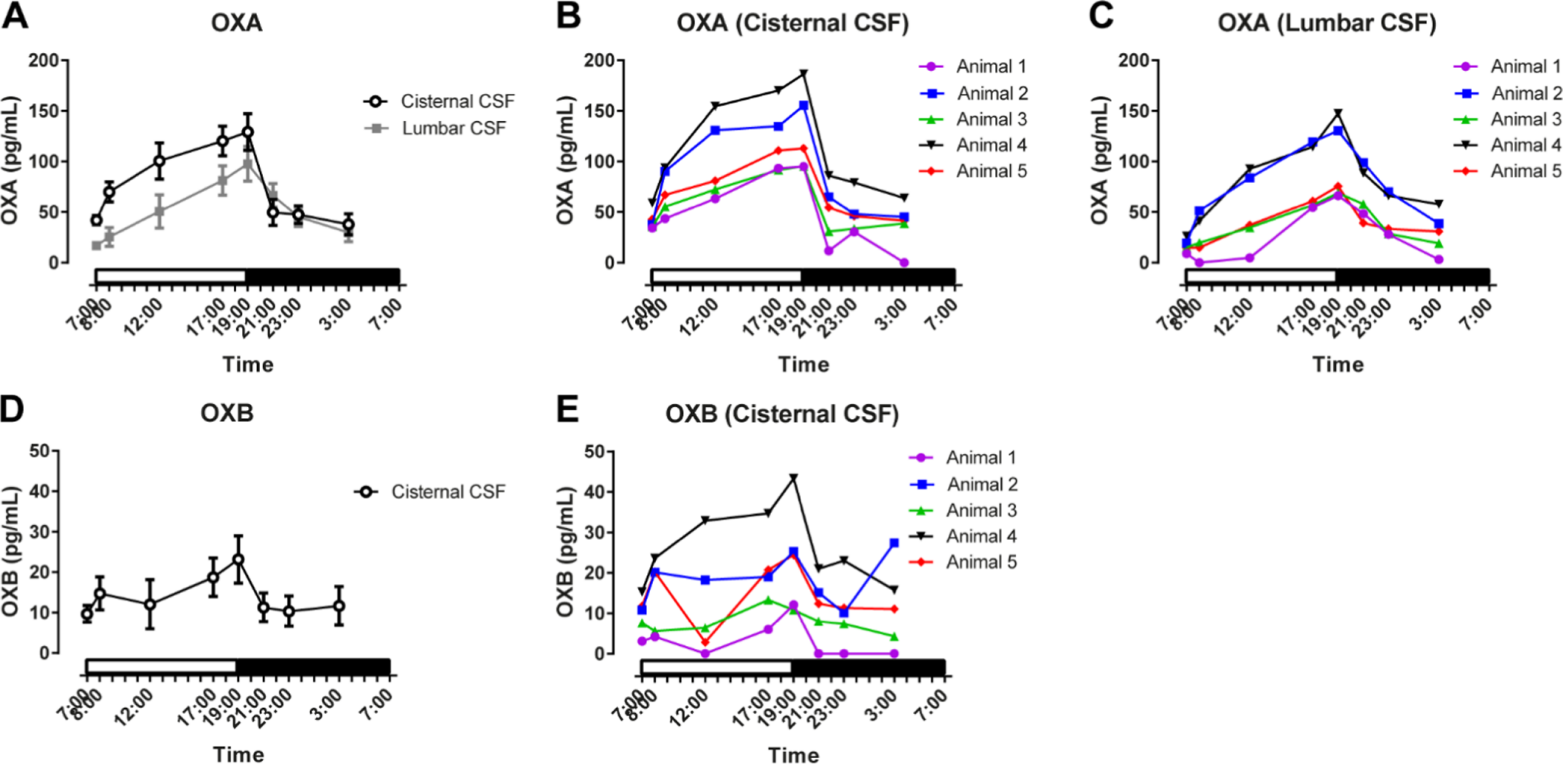
Mean concentrations [mean ± standard error of the mean (SEM), *N* = 5] of (A) OXA and (D) OXB in the CSF. Diurnal fluctuations of OXA in cisternal and lumbar CSF and that of OXB in cisternal CSF were measured. The orexin levels increased during the light period, reached a maximum at the end of the light period, and dropped to the trough level during the dark period. Individual concentrations of OXA in (B) cisternal and (C) lumbar CSF and those of OXB in (E) cisternal CSF. The orexin levels were different between individuals.

The concentrations of OXA and OXB were determined by nanoLC-HRMS in cisternal and lumbar CSF treated with Tween 80 during collection at 3:00, 7:00, 8:00, 12:00, 17:00, 19:00, 21:00, and 23:00. Samples at different time points were collected on different days. The mean concentrations of OXA in cisternal and lumbar CSF ranged from 37.9 to 129.1 pg/mL and 16.9 to 97.8 pg/mL, respectively (Figure 2A). The OXA concentration in the CSF from animal-1 was below the LLOQ in the cisternal sample at 3:00 and the lumbar sample at 8:00, and they were given a value of 0 pg/mL. The OXA level in cisternal CSF sharply increased in the first hour of the light period, gradually increased during the light period, reached a maximum at the end of the light period at 19:00, and then dropped to the trough level of the dark period within 2 h. The lumbar CSF samples contained lower concentrations of OXA with similar diurnal fluctuation patterns to the cisternal CSF, although the decrease rate in the dark period was relatively slower. The lower OXA level in lumbar samples suggested that a portion of OXA was eliminated during CSF migration from the cisterna magna to the lumbar spine. A slower decrease could result from a longer CSF arrival time from the choroid plexus, where CSF was produced. The OXB level in both cisternal and lumbar CSF was also measured. Note that this is the first study to detect OXB in the CSF. The OXB concentration also increased during the light period and decreased in the dark period, with higher levels in the cisternal than the lumbar region (Figures 2D and S3). The concentrations of OXB in four of eight cisternal samples from animal-1 and 28 of 40 lumbar samples were below the LLOQ, and these were given a value of 0 pg/mL. The mean molar ratio of OXA to OXB was 5.3 ± 3.9 (mean ± SD) in cisternal CSF. A higher OXA level can be caused by stabilization of OXA by PTM against enzymatic degradation. The concentrations of OXB were below the LLOQ in lumbar CSF at several time points, especially during the dark period (Figure S3). Further improvement in sensitivity is needed to fully understand OXB fluctuation in lumbar CSF.

**Figure 3:**
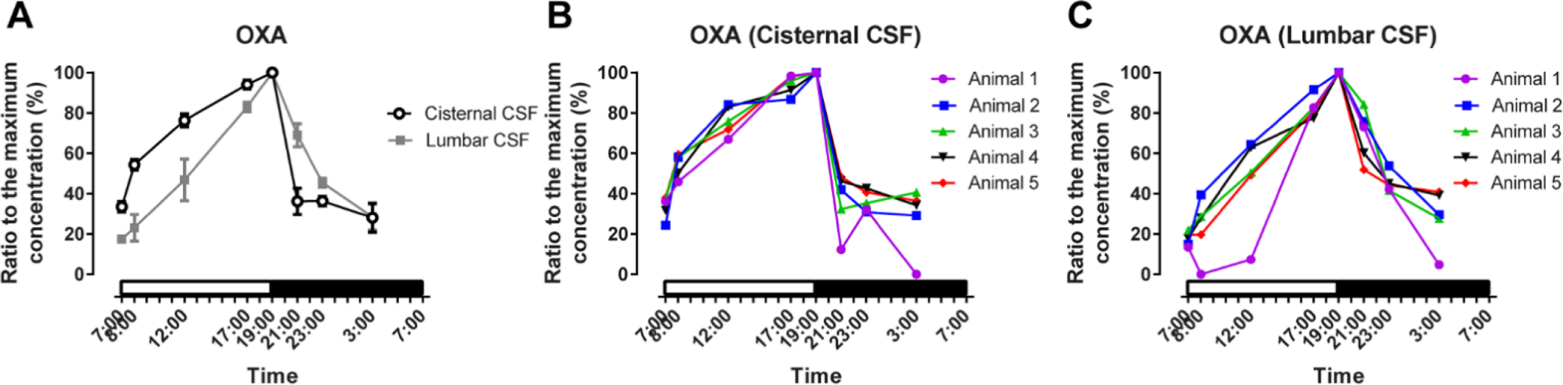
Percentage of OXA relative to the maximum concentration in each animal at various time points. (A) Mean percentage values (mean ± SEM, *N* = 5) of OXA in cisternal and lumbar CSF in all animals. Individual percentage values relative to each maximum concentrations of OXA in (B) cisternal and (C) lumbar CSF. The percentage changes for OXA were similar between individuals.

Interindividual differences in the CSF concentrations of both OXA and OXB were observed (Figures 2B,C,E, and S3). However, the individual monkeys were ranked in similar orders in terms of both OXA and OXB concentrations in cisternal and lumbar CSF. Interestingly, although the individual monkeys showed substantial variation in the absolute OXA concentrations, the percent changes in OXA in cisternal and lumbar CSF at the same time point were similar (Figure 3). The bottom OXA levels at 7:00 were 33.5 and 17.6% of the maximum in cisternal and lumbar CSF, respectively. Note that the samples below LLOQ (cisternal sample taken at 3:00 and lumbar sample taken at 8:00 from animal-1) were given a value of 0.

A previously measured OXA in human lumbar CSF by the RIA method found a smaller range of OXA fluctuation (within ±10% change from the 24 h average).^21^ The different amplitudes of OXA fluctuation in lumbar CSF between monkeys and humans could be caused by the limited number of samples, difference in sample collection and preparation protocols, or the specificity of analytical methods. We believe that our method of nanoLC-HRMS with antiadsorptive additive treatment can be applied to human CSF because it demonstrated sufficient LC separation, highly specific and sensitive HRMS detection, and strong suppression of nonspecific binding. Further investigations are needed to accurately quantify the fluctuations of OXA and OXB in human CSF using the present method. In cisternal CSF, the concentrations of OXA and OXB were the lowest at the beginning of the light period, highest at the end of the light period after a long wake time, and rapidly decreased after starting the dark period. Considering the sleep–wake rhythm, the higher orexin levels during the light period may counteract the increased homeostatic sleep pressure to keep the animal awake. Immediately after starting the dark period, the rapid decrease in orexin levels could support efficient sleep induction. The diurnal fluctuation pattern of orexins in cisternal CSF revealed in this study could help understand the ideal activation pattern of orexin signaling by OX2R agonists in individuals with NT1.

### Interday Variation of OXA and OXB Concentrations in Monkey CSF

**Figure 4:**
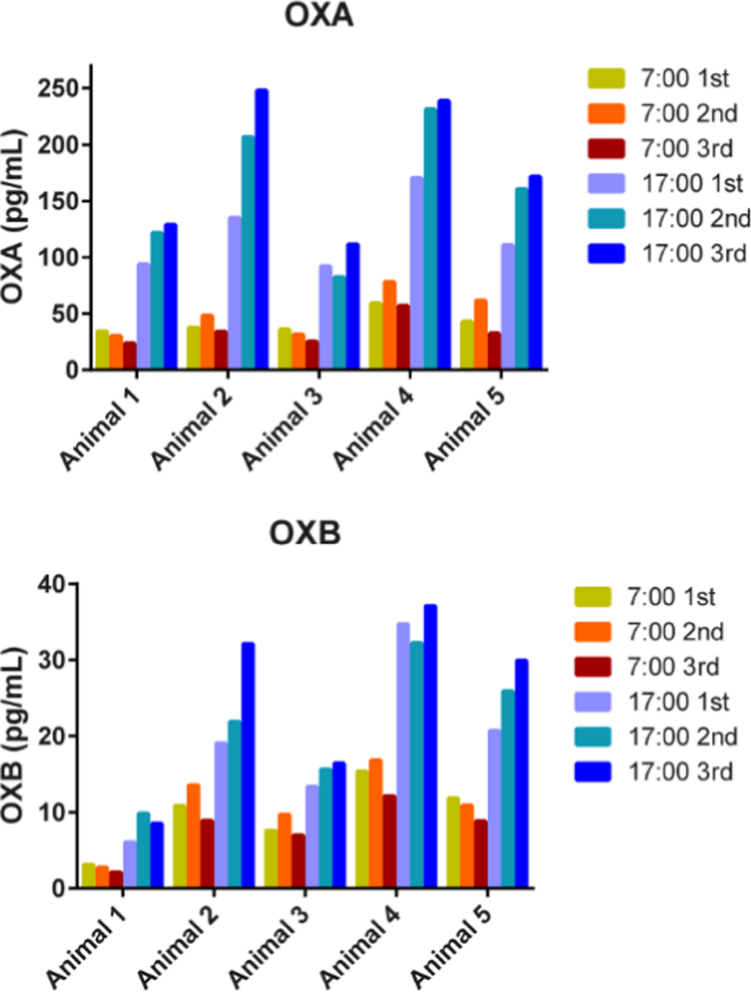
Interday variation in the concentrations of OXA and OXB in cisternal CSF collected from the same five animals at two time points on three different days (at intervals of ∼1 week and ∼10 months). The mean % CV (*N* = 5) values were within 16.1%, demonstrating acceptable interday variation.

Interday variation of OXA and OXB levels was assessed using cisternal CSF collected from the same five individual animals on three different days (Figure 4). The samples at 7:00 were collected with intervals of 5 days and 10 months, and those at 17:00 were collected with intervals of 11 days and 10 months. Similar to the trends in Figure 2, the OXA and OXB levels were different among individuals with the mean concentrations ranging from 29.4 to 64.6 pg/mL (7:00) and 95.1 to 213.3 pg/mL (17:00) for OXA, and 2.9 to 14.8 pg/mL (7:00) and 8.1 to 34.7 pg/mL (17:00) for OXB. In contrast, the mean % CVs of orexin concentrations in each animal were much smaller: 15.0% (7:00) and 16.1% (17:00) for OXA, and 13.1% (7:00) and 11.6% (17:00) for OXB. Considering that the % CV values include the analytical variability shown in Table 2 (% CV < 14.0%), our data demonstrated acceptable interday variation of the OXA and OXB concentrations, even for samples at the 10-month interval. In other words, individual monkeys maintained their OXA and OXB levels over a long duration. These results support that CSF collection on different days is an alternative method to serial collection on the same day. Therefore, our data obtained with this sampling approach to evaluate diurnal fluctuations should be accurate and reliable.

### Comparison of OXA and OXB in the CSF Collected with and without Antiadsorptive Additives

**Figure 5:**
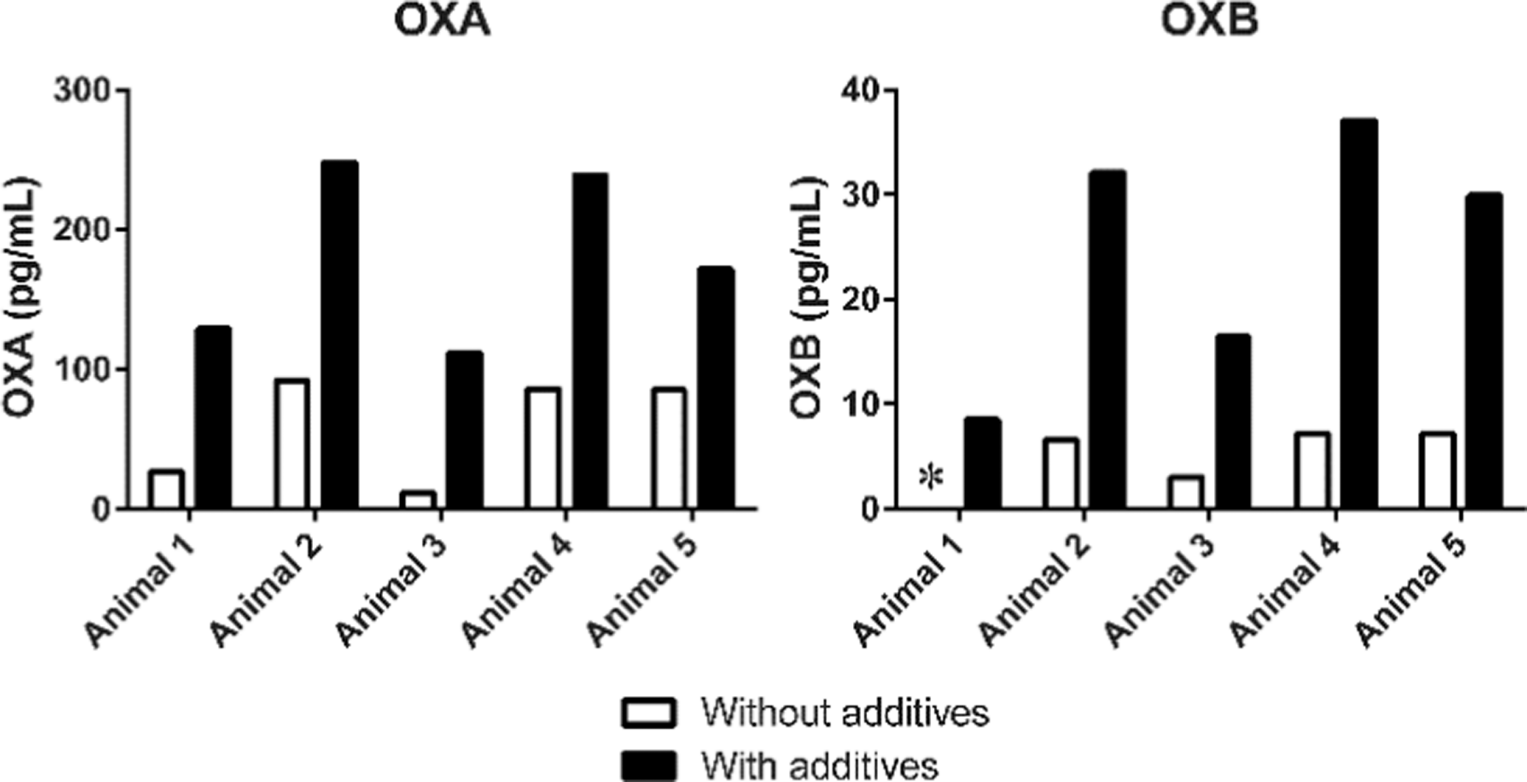
Recovery of OXA and OXB in cisternal CSF samples collected with and without 20 mM citric acid and 0.1% (w/v) Tween 80 (final concentrations). Samples collected without additives demonstrated low recoveries of 10.3–49.8% for OXA and 18.0–24.0% for OXB. *< LLOQ.

To assess the effectiveness of antiadsorptive additives, we compared the concentrations of OXA and OXB in cisternal CSF collected with and without 20 mM citric acid and 0.1% (w/v) Tween 80 (final concentrations) (Figure 5). The samples were collected at the same time points on different days. Low concentrations of OXA and OXB were observed in the samples without additives, similar to results of spike recovery (Table 2). The ion suppression by antiadsorptive additives was evaluated using the peak area ratios of IS in the CSF samples with and without 20 mM citric acid and 0.1% (w/v) Tween 80 (final concentrations). The ratios (with/without additives) were 0.7 for OXA and 0.6 for OXB. The concentration ratios between samples without and with the antiadsorptive additives ranged from 10.3 to 49.8% (mean 30.7%, *N* = 5) for OXA and from 18.0 to 24.0% (mean 20.5%, *N* = 4) for OXB. In the sample collected from animal-1 without additives, the OXB level was below the LLOQ and therefore excluded from the ratio calculation. The large variability in the ratio of OXA made it difficult to correct the concentration using a fixed coefficient. These results show that CSF samples collected without antiadsorptive additives were not appropriate for OXA and OXB determination. We hypothesize that severe nonspecific binding of OXA and OXB could cause the variability of OXA concentrations and undetectable OXB reported previously for human CSF. Further investigations should be conducted using newly collected CSF samples with appropriate reagents added for each sample.

## Conclusions

We established a novel method to precisely measure OXA and OXB levels in monkey CSF. Antiadsorptive additives—citric acid and Tween 80—were added during sample collection and preparation steps to prevent the nonspecific binding of orexins. Then, OXA and OXB levels in the CSF were measured using the nanoLC-HRMS approach. Employing this method, we revealed diurnal fluctuations of OXA and OXB in cisternal and lumbar CSF samples from monkeys. Better characterization of orexin levels, including diurnal fluctuation under various conditions, would improve our understanding of orexin physiology, as well as help to establish the ideal pharmacokinetic pattern of OX2R agonists for treating related diseases. Further studies of both OXA and OXB levels using this method should provide valuable information on the role that orexins play in human health and diseases.

## Methods

### Reagents

Synthetic standards for OXA and OXB were purchased from Peptide Institute (Osaka, Japan). Stable isotope-labeled OXA and OXB were used as ISs for nanoLC-HRMS: OXA with a mass shift of 42 Da by six [^13^C_6_, ^15^N]Leu (Phoenix Pharmaceuticals, Burlingame, CA) and OXB with a mass shift of 17 Da by one [^13^C_6_, ^15^N]Leu and one [^13^C_6_, ^15^N_4_]Arg (Scrum Inc., Tokyo, Japan). OXA metabolite with amide hydrolysis at the C-terminal leucine (OXA-Leu33-OH) was obtained from Scrum Inc. Antiadsorptive reagents used were 20 mM citric acid and 0.1% (w/v) Tween 80 (final concentrations). All other chemicals, reagents, and solvents were analytical grade.

### Preparation of Stock Solutions, Calibration Curves, Quality Control Samples, and Blank Samples

Eppendorf Protein LoBind tubes (Eppendorf, Tokyo, Japan) were used for sample preparation. Synthetic orexins without isotope labeling were dissolved in dimethyl sulfoxide (DMSO) at 100 μg/mL as OXA and OXB stock solutions. The isotope-labeled ones were dissolved in DMSO at 20 μg/mL as IS stock solutions. The stock solutions of OXA, OXB, and ISs were stored at −30 °C until use.

Calibration standards of OXA and OXB corresponding to additional 2.5, 5, 20, 25, 50, 100, and 250 pg/mL were freshly prepared using surrogate or real matrices by serial dilution of the corresponding stock solutions with DMSO. Working IS solutions at 500 pg/mL were prepared by diluting the IS stock solutions with a mixture of 0.1% (w/v) bovine serum albumin and 20 mM citric acid and 0.1% (w/v) Tween 80 (final concentrations) in Dulbecco’s phosphate-buffered saline (PBS) as the surrogate matrix. The surrogate matrix was used as a blank matrix for the calibration curve, SQC samples for method qualification, and blank samples. The SQC samples were prepared by adding working solution of a mixture of OXA and OXB at the final concentrations of 2.5 pg/mL (LLOQ), 7.5 pg/mL (LQC), 120 pg/mL (MQC), and 200 pg/mL (HQC). RQC samples, which were monkey CSF containing 20 mM citric acid and 0.1% (w/v) Tween 80, were also prepared to confirm the parallel between the surrogate and real matrices. The spiked RQC samples were prepared by adding OXA and OXB corresponding to 25 and 125 pg/mL, respectively. The freeze–thaw stability, stability at −80 °C, and stability at room temperature were determined using the spiked RQC samples with additional OXA and OXB corresponding to 25 and 125 pg/mL, respectively. Stability was evaluated based on the difference from the initial sample. The processed sample stability was evaluated using three replicates of real matrix. The samples were analyzed with calibration samples, and stored in an autosampler for at least 48 h. The stored samples were reinjected with freshly processed calibration samples. The processed sample stability was evaluated to compare the back-calculated concentrations.

### Recovery during CSF Collection and Tube Transfer

Recovery of OXA and OXB during CSF sample collection was evaluated with samples prepared by the following procedure (Figure S2). The samples were prepared in triplicate. Monkey CSF (297 μL) was mixed with OXA and OXB mixture (3 μL of 10 ng each/mL in DMSO) in a LoBind tube. The whole sample was removed from the tube using an injection needle (25G, Terumo Corporation, Tokyo, Japan) and a syringe (Terumo Corporation, Tokyo, Japan). Then, 1 M citric acid (6 μL) and 10% (w/v) Tween 80 (3 μL) were added to the empty tube to reach the final concentrations of 20 mM and 0.1%. The sample was returned from the syringe to the tube. The tube and syringe were then rinsed by pipetting five times with the CSF containing 20 mM citric acid and 0.1% (w/v) Tween 80. Finally, the whole sample was transferred to another tube. Recovery during tube transfer was assessed after repeating the procedure three times with monkey CSF containing 20 mM citric acid and 0.1% (w/v) Tween 80. The reference sample was prepared without syringe transfer, pipetting, or tube transfer. Recovery was calculated based on the quantitative values, which were the total values of endogenous and spiked orexins, in the testing samples to that in the reference sample.

### Sample Preparation for nanoLC-HRMS

LoBind tubes were used for preparing the nanoLC-HRMS samples. The IS working solution (60 μL) was mixed with a CSF, calibration curve, SQC, or RQC (50 μL) sample. Samples for the calibration curves were added with freshly prepared calibration standards (5 μL) corresponding to 2.5, 5, 20, 25, 50, 100, and 250 pg/mL, while DMSO (5 μL) was added to other samples. The samples were mixed with 10% (v/v) phosphoric acid (20 μL) and then centrifuged at 18,360*g* for 5 min at 4 °C. The supernatant (120 μL) was loaded into an HLB μElution plate and washed with 200 μL of methanol/water mixture (5:95, v/v). Orexins and ISs were eluted with 200 μL of acetonitrile/water/formic acid (80:20:0.1, v/v/v). After centrifugation at 4283*g* for 5 min at 4 °C, the supernatant (180 μL) was evaporated to dryness under a stream of nitrogen gas at room temperature. The precipitates were reconstituted in 40 μL of acetonitrile/water/trifluoroacetic acid (TFA) (4:96:0.01, v/v/v). After centrifugation at 12,000*g* for 10 min at 4 °C, the supernatant (5 μL) was used for nanoLC-HRMS.

### NanoLC-HRMS

NanoLC-HRMS was performed on an EASY-nLC 1200 system (Thermo Fisher Scientific, Inc., Waltham, MA) and a Q Exactive HF-X Orbitrap mass spectrometer (Thermo Fisher Scientific) equipped with an electrospray ionization interface. The reconstituted samples were kept in an autosampler at 10 °C, and then, 5 μL of each sample was loaded onto an Acclaim PepMap 100 C18 trap column (3 μm, 0.075 mm i.d. × 20 mm, Thermo Fisher Scientific) coupled with an NTCC-360/75–3–15 analytical capillary column (3 μm, 0.075 mm i.d. × 150 mm, Nikkyo Technos, Tokyo, Japan) at room temperature. TFA/water (0.01:100, v/v) and TFA/water/acetonitrile (0.01:20:80, v/v/v) were used as mobile phases A and B, respectively. The total flow rate was 300 nL/min, and the following gradient elution was used: 0–30 min, 5–30% B; 30–38 min, 30–50% B; 38–45 min, 50–95% B; and 45–55 min, 95% B. The mass spectra were obtained in the positive ion mode under the following conditions: resolving power of 60,000 (full width at half maximum), automatic gain control (AGC) target of 200,000, spray voltage of 2.00 kV, capillary temperature of 275 °C, and funnel RF level of 45.0. The precursor ions selected with an isolation width of *m*/*z* 4.0 underwent higher-energy collision dissociation at a normalized collision energy of 20. Survivor-SIM analysis was employed for higher sensitivity.^22^ PRM was conducted with the same conditions expect for resolving power of 120,000, AGC target of 1,000,000, and normalized collision energy of 27 for OXA and 30 for OXB. The following transitions were monitored for quantification: 1187.24411 → 1187.91011 for OXA, 1201.27843 → 1201.2780 for OXA IS, 966.87003 → 967.20176 for OXB, and 972.54517 → 972.8734 for OXB IS. Isotopic ions were selected to achieve higher sensitivity. All of the ions were [M + 3H]^+^.

### Animals

The experiments were approved by the Institutional Animal Care and Use Committee of Shonan Research Center, Takeda Pharmaceutical Company Limited. Five male cynomolgus monkeys (aged 4–5 years, Hamri Co., Ltd., Ibaraki, Japan) were used in this study. Each cage housed a single animal, and two cages were connected to allow the animals to interact with each other. Starting from 1 week before a series of CSF collections, the animals were single-housed for 1 month. Then, two cages were connected again for at least 1 week before another series of CSF collections. Throughout the experiments, one animal (animal-3) was housed singly because the total number of five is odd. The monkeys were kept under a 12-h light/dark cycle with light between 7:00 and 19:00. Food was provided daily in the morning.

### CSF Collection

The animals were anesthetized in the home cage by intramuscular injection of 10 mg/kg ketamine (KETALAR, Daiichi Sankyo Co., Ltd., Tokyo, Japan). CSF was collected from anesthetized animals only once a day, and there was an interval of more than 48 h before the next collection. CSF samples were collected via either cisterna magna puncture or lumbar puncture.

For cisterna magna puncture, CSF was sampled with an injection needle (25G, Terumo Corporation, Tokyo, Japan) and a syringe (1 mL, Terumo Corporation, Tokyo, Japan). Injection needle consists of a needle and a needle hub. The needle is made of stainless steel and the needle hub is made of polypropylene. For syringe, the parts that contact CSF samples are the rubber stopper on the plunger and the barrel. The rubber stopper is made of elastomer and the barrel is made of polypropylene. The volume of collected CSF sample was approximately 250 μL for diurnal fluctuation experiment. Orexin levels in CSF samples collected at 7:00 and 17:00 were also used as orexin levels in the CSF of first collection in interday variation experiment. For other collections, the volume of CSF sample was approximately 500 μL. Tween 80 (1%, v/v) in PBS was generally added to the collected CSF (Tween 80/CSF = 1:100, v/v) to reach a final concentration of 0.01%. However, for the second and third collection in the interday variation experiment, 10% Tween 80 (v/v) in PBS and 1 M citric acid were added to the collected CSF (Tween 80/citric acid/CSF = 1:2:100, v/v) to reach a final concentration of 0.1% and 20 mM, respectively, instead. Pipetting was conducted in the collection tube (Eppendorf Protein LoBind) with the syringe and injection needle used for CSF collection (Figure S2).

For lumbar puncture, an injection needle (25G, Terumo Corporation, Tokyo, Japan) was inserted between L3 and L5, and CSF leaked through the injection needle was sampled into a collection tube. The volume of collected CSF sample was approximately 250 μL. Tween 80 (1%, v/v) was added to the CSF (Tween 80/CSF = 1:100, v/v) to reach a final concentration of 0.01%, and pipetting was conducted in the collection tube with the used injection needle and a brand new syringe. All collected CSF samples were kept on ice until centrifugation (4 °C, 18,000*g*, 1 min). The supernatant was transferred to a new collection tube and stored in a freezer at −80 °C until use. The time interval between sample collection and placement in the freezer was within 1 h.

### Diurnal Fluctuations of Orexin Levels

CSF was collected via cisterna magna puncture, followed by lumbar puncture at 3:00, 7:00, 8:00, 12:00, 17:00, 19:00, 21:00, and 23:00. For nighttime CSF collection (3:00, 19:00, 21:00, and 23:00), the animals were anesthetized in the dark, blindfolded, and then transferred to a bright room for sample collection to mitigate the potential effect of light on CSF orexin levels. It took 7 weeks to collect all samples used to analyze diurnal fluctuations.

### Interday Variation of Orexin Levels

CSF was collected via cisterna magna puncture. Orexin levels in CSF samples collected at 7:00 and 17:00 in the diurnal fluctuation experiment were also used as orexin levels in the CSF of first collection in this experiment. For samples collected at 7:00, the interval was 10 months between the first and second collection and 11 days between the second and third collection. For CSF collected at 17:00, the interval was 10 months between the first and second collection and 5 days between second and third collection.

### Recovery of Orexin with and without Antiadsorptive Additives

CSF was collected via cisterna magna puncture at 17:00. Orexin levels in CSF samples collected at the third collection in the interday variation experiment were also used as orexin levels in CSF with 20 mM citric acid and 0.1% (w/v) Tween 80 (final concentrations) in this experiment. After the third collection in the interday variation experiment, CSF samples without antiadsorptive additives were collected with a 3-month interval.

### Statistics and Data Analysis

Xcalibur 4.1 and TraceFinder 4.1 (Thermo) were used for data acquisition, peak detection, integration, calibration, and quantification. Results of CSF analysis are expressed as the mean ± standard error of the mean (SEM). Graphs were created using GraphPad (v6) software.

## Supporting Information Available

Additional experimental details of CSF collection and recovery testing and OXB concentrations in lumbar CSF (PDF)

## Abbreviations

% CV: coefficient of variation
% RE: percent mean relative error
CSF: cerebrospinal fluid
ECL: electrochemiluminescence
HQC: higher quality control
HRMS: high-resolution mass spectrometry
IS: internal standard
KO: knock-out
LC–MS/MS: liquid chromatography–tandem mass spectrometry
LLOQ: lower limit of quantification
LQC: lower quality control
MQC: middle quality control
nanoLC-HRMS: nanoflow liquid chromatography–high-resolution mass spectrometry
NT1: narcolepsy type 1
OX1R: orexin 1 receptor
OX2R: orexin 2 receptor
OXA: orexin A
OXB: orexin B
QC: quality control
RIA: radioimmunoassay
RQC: real matrix quality control
SEM: standard error of the mean
SIM: survivor-selected ion monitoring
SQC: surrogate matrix quality control
