# Supplementary material for: Diurnal Fluctuations of Orexin-A and -B in Cynomolgus Monkey Cerebrospinal Fluid Determined by a Novel Analytical Method Using Antiadsorptive Additive Treatment Followed by Nanoflow Liquid Chromatography–High-Resolution Mass Spectrometry
Source: ACS Chem Neurosci. 2023 Jan 31;14(4):609–18. doi: 10.1021/acschemneuro.2c00370 (PMC9936545; doi:10.1021/acschemneuro.2c00370)
Supplement: Supplementary file 1 — cn2c00370_si_001.pdf [file cn2c00370_si_001.pdf]

Diurnal Fluctuations of Orexin-A and -B in Cynomolgus Monkey Cerebrospinal Fluid Determined by a Novel Analytical Method Using Antiadsorptive Additive Treatment Followed by Nanoflow Liquid Chromatography-High-Resolution Mass Spectrometry

Naohiro Narita,[§,‡] Ryuji Yamada,[//,‡] Masaaki Kakehi,[§] and Haruhide Kimura*[,//]

[§] Drug Metabolism and Pharmacokinetics Laboratory, Research, Takeda Pharmaceutical Company Limited, 26-1 Muraoka-Higashi 2-chome, Fujisawa, Kanagawa, 251-8555, Japan.

[//] Neuroscience Drug Discovery Unit, Research, Takeda Pharmaceutical Company Limited, 26-1 Muraoka-Higashi 2-chome, Fujisawa, Kanagawa, 251-8555, Japan.

[‡]These authors contributed equally to this work.

* E-mail: haruhide.kimura@takeda.com

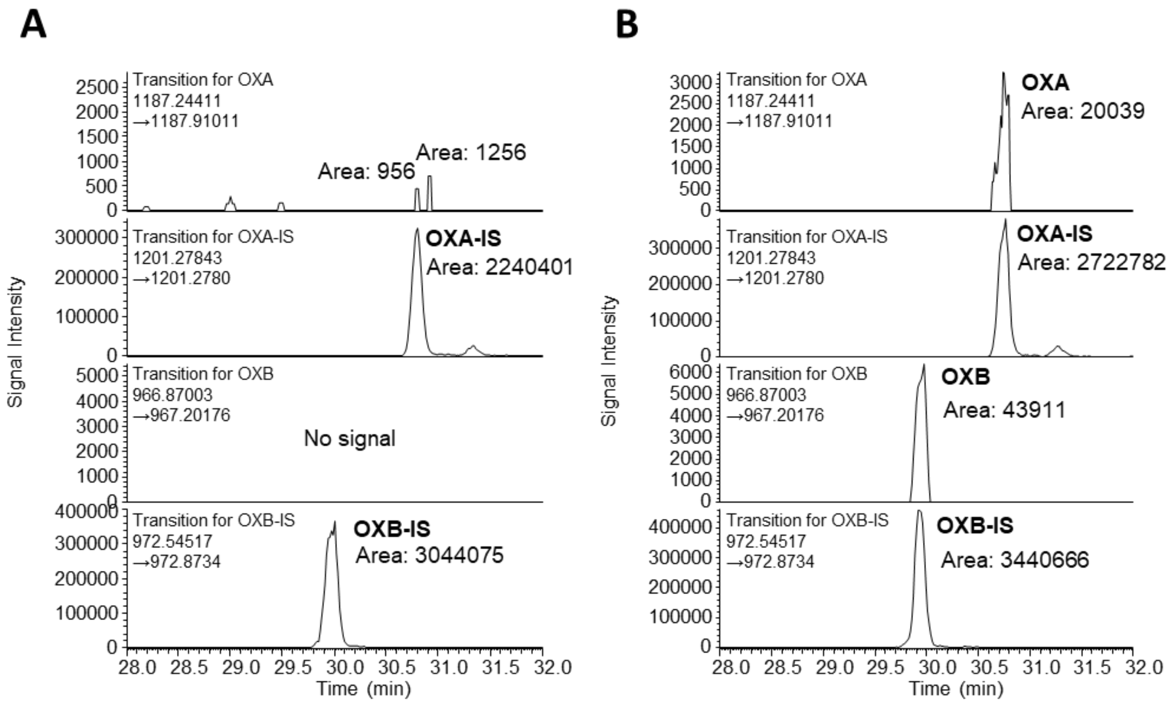

Figure S1

Representative chromatograms of OXA, OXB, and ISs from (A) zero calibrator and (B) calibration sample at LLOQ 2.5 pg/mL. The analyte signals of the LLOQ sample was greater than 9 times the signal of zero calibrator sample, indicating that ISs had no impact on the quantitation of unlabeled orexins. The following transitions were monitored for quantification: 1187.24411→1187.91011 for OXA, 1201.27843→ 1201.2780 for OXA IS, 966.87003→967.20176 for OXB, 972.54517→972.8734 for OXB IS. All the ions were $[M+3H]^+$.

Diurnal Fluctuations of Orexin-A and -B in Cynomolgus Monkey Cerebrospinal Fluid Determined by a Novel Analytical Method Using Antiadsorptive Additive Treatment Followed by Nanoflow Liquid Chromatography-High-Resolution Mass Spectrometry

Naohiro Narita,[§,‡] Ryuji Yamada,[//,‡] Masaaki Kakehi,[§] and Haruhide Kimura*[,//]
[§]Drug Metabolism and Pharmacokinetics Laboratory, Research, Takeda Pharmaceutical Company Limited, 26-1 Muraoka-Higashi 2-chome, Fujisawa, Kanagawa, 251-8555, Japan.
[//]Neuroscience Drug Discovery Unit, Research, Takeda Pharmaceutical Company Limited, 26-1 Muraoka-Higashi 2-chome, Fujisawa, Kanagawa, 251-8555, Japan.
[‡]These authors contributed equally to this work.
* E-mail: haruhide.kimura@takeda.com

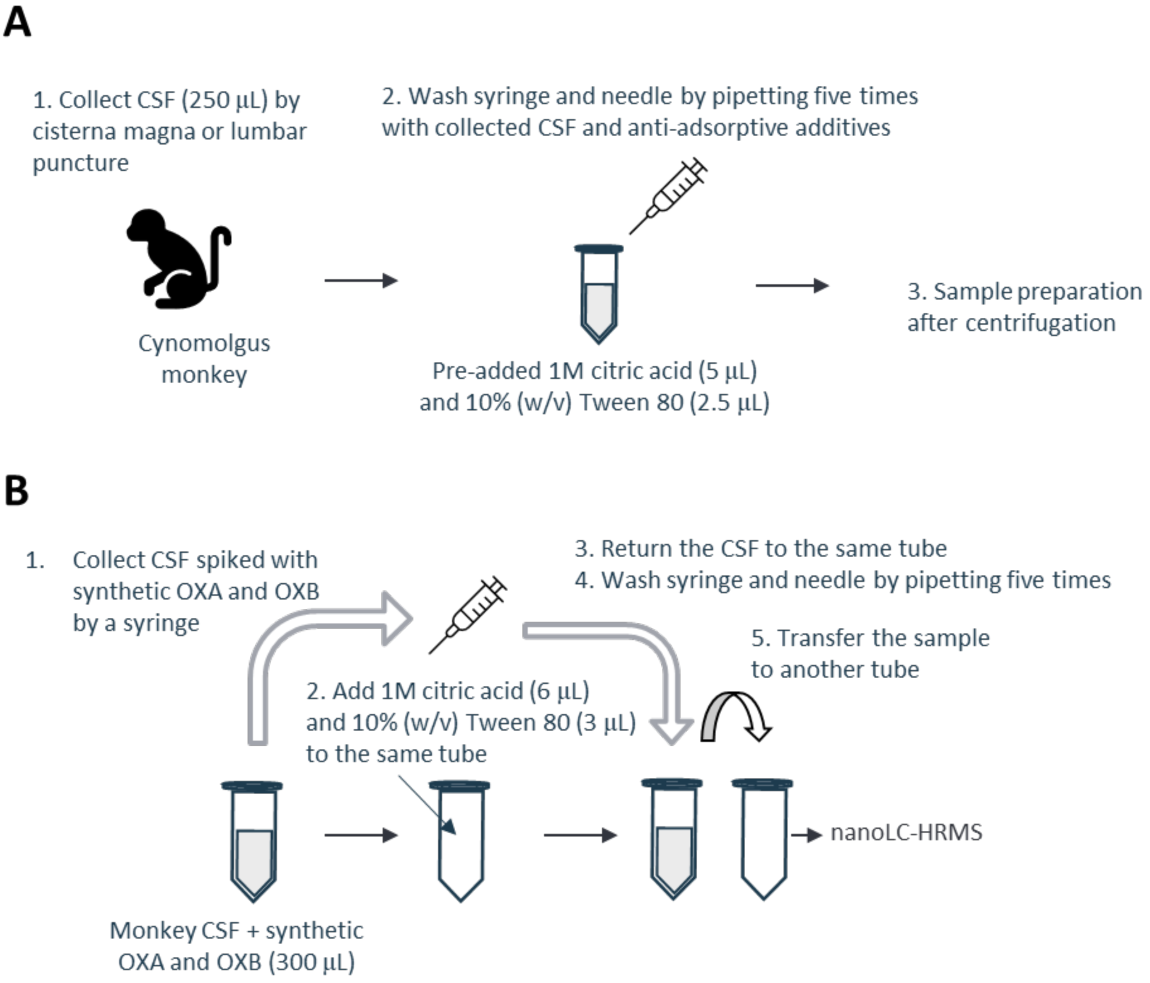

Figure S2

CSF collection from cynomolgus monkey (A). Eppendorf Protein LoBind® tubes (1.5mL) were used for sample preparation. The syringe and needle used for CSF collection were washed by pipetting five times with collected CSF including anti-adsorptive additives to prevent non-specific adsorption to the instruments. Recovery test during syringe and tube transfer (B). Eppendorf Protein LoBind® tubes (1.5mL) were used for sample preparation. A tube containing neat CSF spiked with the standards for OXA and OXB was prepared. The spiked CSF was collected from the tube using a needle and a syringe. At this point, some portions of OXA and OXB were adsorbed to the tube. Then, 6 µL of 1M citric acid and 3 µL of 10% (w/v) Tween 80 were added to the tube to achieve the final concentrations of 20 mM citric acid and 0.1% Tween 80, and the 300 µL of spiked CSF was returned from the syringe to the tube. The tube, the needle, and the syringe were rinsed by pipetting five times with CSF containing citric acid/Tween 80. Sufficient recovery of OXA and OXB indicated that orexins adsorbed to syringes and tubes could be recovered by the addition of 20 mM citric acid and 0.1% Tween 80.

Diurnal Fluctuations of Orexin-A and -B in Cynomolgus Monkey Cerebrospinal Fluid Determined by a Novel Analytical Method Using Antiadsorptive Additive Treatment Followed by Nanoflow Liquid Chromatography-High-Resolution Mass Spectrometry

Naohiro Narita,[§,‡] Ryuji Yamada,[//,‡] Masaaki Kakehi,[§] and Haruhide Kimura*[,//]

[§] Drug Metabolism and Pharmacokinetics Laboratory, Research, Takeda Pharmaceutical Company Limited, 26-1 Muraoka-Higashi 2-chome, Fujisawa, Kanagawa, 251-8555, Japan.

[//] Neuroscience Drug Discovery Unit, Research, Takeda Pharmaceutical Company Limited, 26-1 Muraoka-Higashi 2-chome, Fujisawa, Kanagawa, 251-8555, Japan.

[‡]These authors contributed equally to this work.

* E-mail: haruhide.kimura@takeda.com

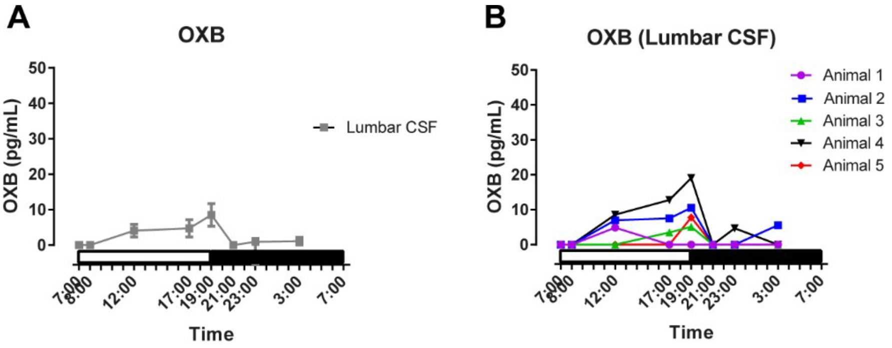

Figure S3

(A) Mean concentrations (mean ± SEM, $N = 5$) and (B) individual concentrations of OXB in cisternal and lumbar CSF. OXB level in lumbar CSF increased during the light period, reached a maximum at the end of the light period, and dropped to trough level during the dark period. Note that there were many missing values; 28 of 40 lumbar samples were below the LLOQ. Further improvement in sensitivity is needed to fully understand OXB fluctuation in lumbar CSF.
